# Whole genome sequence of *Staphylococcus aureus* strain RMI-014804 isolated from pulmonary patient sputum via next-generation sequencing technology

**DOI:** 10.5808/gi.23024

**Published:** 2023-09-27

**Authors:** Ayesha Wisal, Asad Ullah, Waheed Anwar, Carlos M. Morel, Syed Shah Hassan

**Affiliations:** 1 Department of Chemistry, Islamia College Peshawar, Peshawar, KP 25000, Pakistan; 2 Department of Pulmonology, Rehman Medical Institute, Peshawar, KP 25000, Pakistan; 3 Centre for Technological Development in Health (CDTS), Oswaldo Cruz Foundation (Fiocruz), Building "Expansão", 8th floor room 814, Av. Brasil 4036 - Manguinhos, Rio de Janeiro, RJ 21040-361, Brazil; 4 Jamil–ur–Rahman Center for Genome Research, Dr. Panjwani Center for Molecular Medicine and Drug Research, International Center for Chemical and Biological Sciences, University of Karachi, Karachi 75270, Pakistan

**Keywords:** genome sequencing, Illumina MiSeq, nosocomial infections, phylogenetic analysis, *Staphylococcus aureus*

## Abstract

Nosocomial infections, commonly referred to as healthcare-associated infections, are illnesses that patients get while hospitalized and are typically either not yet manifest or may develop. One of the most prevalent nosocomial diseases in hospitalized patients is pneumonia, among the leading causes of mortality and morbidity. Viral, bacterial, and fungal pathogens cause pneumonia. More severe introductions commonly included *Staphylococcus aureus*, which is at the top of bacterial infections, per World Health Organization reports. The staphylococci, *S. aureus*, strain RMI-014804, mesophile, on-sporulating, and non-motile bacterium, was isolated from the sputum of a pulmonary patient in Pakistan. Many characteristics of *S. aureus* strain RMI-014804 have been revealed in this paper, with complete genome sequence and annotation. Our findings indicate that the genome is a single circular 2.82 Mbp long genome with 1,962 protein-coding genes, 15 rRNA, 49 tRNA, 62 pseudogenes, and a GC content of 28.76%. As a result of this genome sequencing analysis, researchers will fully understand the genetic and molecular basis of the virulence of the *S. aureus* bacteria, which could help prevent the spread of nosocomial infections like pneumonia. Genome analysis of this strain was necessary to identify the specific genes and molecular mechanisms that contribute to its pathogenicity, antibiotic resistance, and genetic diversity, allowing for a more in-depth investigation of its pathogenesis to develop new treatments and preventive measures against infections caused by this bacterium.

## Introduction

Pneumonia is an inflammatory lung illness mainly affecting the alveoli [1,2]. It is one of the leading causes of death and morbidity worldwide. Every year, roughly 450 million people are impacted, with 4 million (7% of total mortality) projected fatalities worldwide [3,4]. According to one study, pneumonia is the eighth leading cause of death in the United States and the fourth leading cause of death worldwide [5]. According to the World Health Organization, with 16% of all fatalities in children under five occurring from pneumonia, it is the leading cause of mortality in this age group globally [3]. Every year, around 200 million cases of both adults and children occur. The illness is five times more prevalent in developing nations than in wealthy countries [4].

Viruses, bacteria, and fungi are just a few microorganisms that can cause pneumonia, an acute inflammatory disease of the lung parenchyma [6]. *Staphylococcus aureus* is the cause of severe pneumonia [7]. Staphylococcal pneumonia induces alveolar and capillary inflammation, resulting in blood stasis. The capillary membrane of the alveoli degrades, resulting in exudative pleural effusion and atelectasis [8].

Many clinical signs are brought on by *S. aureus*, a frequent nosocomial and community-acquired infection. The skin and mucous membranes of various hosts, such as people, poultry, cattle, birds, and sheep, are where the pathogen lives or momentarily colonize [9]. An opportunistic bacteria called *S. aureus* infects 20%–40% of people and lives asymptomatically in the skin flora, nasopharynx, throat, digestive system, lower female reproductive tract, and anterior nares [10]. It's the leading cause of skin and soft tissue infections. It enters the circulation through subcutaneous tissues and affects heart valves and tissues [11].

In chronic wounds such as (surgical site and traumatic wounds), ulcers like foot ulcers(diabetic and venous), and pressure ulcers, *S. aureus* is usually found as a commensal [12]. The most severe disease that *S. aureus* may cause is pneumonia, the leading cause of death worldwide. Because of the illness's rising prevalence and quick transmission within the afflicted animal population, interventions must be implemented to prevent disease spread and safeguard nearby species.

### Classification and features

*S. aureus* is a gram-positive, non-sporulating, non-motile, spherical member of the Staphylococcaceae family of bacteria [13]. The bacterium was first discovered in 1881 by Sir Alexander Ogston from pus in a surgical abscess and named *Staphylococcus* after its clustered appearance with a diameter of 0.5–1.5 µm [14]. These organisms can grow aerobically or anaerobically (facultative) by aerobic respiration or by fermentation [15] and at a temperature between 18°C and 40°C [16]. The optimum pH for metabolism ranges from 7.0 to 7.5 [17]. After initially growing sporadically, strain RMI-014804 forms round, raised, opaque, yellow to golden colonies on an agar surface [18]. *S. aureus* is classified into different biotypes according to color and growth [19]. The thick protective layer of the *S. aureus* cell wall has an amorphous appearance. The core of the cell wall, which makes up around 50% of its bulk, is peptidoglycan [20]. Teichoic acid, which makes up approximately 40% of the mass of the cell wall and is a group of phosphate-containing polymers, is another component of the cell wall. Teichoic acids are classified into two types: wall teichoic acids and lipoteichoic acids. Wall teichoic acids are layers of lengthy anionic peptidoglycan polymers comprised mostly of glycerol phosphate, glucosyl phosphate, or ribitol phosphate repetitions. The teichoic acids in the wall are covalently linked to lipoteichoic acids, which are bonded to the head groups of membrane lipids [21].

To cling to plasma and extracellular matrix, *S. aureus* has many unique adhesions on its surface that bind with a range of host proteins, including fibronectin, fibrinogen, collagen, vitronectin, and laminin [22]. These adhesions are MSCRAMMs (microbial surface components recognizing adhesive matrix molecules) [23]. All *S. aureus* isolates produce coagulase, catalase, and an extracellular cell clumping factor; some bacteria create capsules [24].

## Methods

### Selection of genomes

The bacterial strains were chosen due to their rapidly developing resistance to antibiotics for widespread clinical usage, the discovery of therapeutic targets, and the development of novel medicines.

### Sample collection

A bacterial sample was collected from the sputum of a pulmonary patient at Rehman Medical Institute (RMI) in Peshawar, Pakistan. The isolate was identified using standard procedures and tests such as gram staining, catalase, coagulase, and DNase [25]. The Centre of Genomic Sciences in RMI Peshawar, KPK, Pakistan, undertook further analysis, sequencing, and annotation.

### Growth condition and DNA isolation

With NaCl concentrations of up to 15%, *S. aureus* may thrive at temperatures ranging from 15°C to 45°C. Mannitol salt agar with 7.5% NaCl was used as a selective medium. *S. aureus* was grown for 24 h in a 37°C incubator with a 200-rpm shaker on rich media such as tryptic soy agar, brain heart infusion, and Luria Bertani. To suspend pure colonies in 300 µL, tent buffer (10 mM Tris-HCl, 0.1 M NaCl, 1 mM EDTA, 5% [v/v] Triton X100, pH 8.0) was employed. After boiling at 100°C, the cell suspension was centrifuged. The supernatant fluid was transferred to a new sterile tube. The supernatant was treated for 20 min at –20°C with cold 95% ethanol. The solution was then centrifuged. The DNA templates were maintained at –20°C after being dissolved in 50 µL of sterile distilled water. A NanoDrop ND-1000 spectrophotometer assessed the solution's concentration and purity.

### DNA extraction and library preparation

For sequencing genomic DNA was extracted using the Qiagen Kit (Qiagen, Valencia, CA, USA) according to the manufacturer's instructions, quantified by a nano-drop (Thermo Fisher Scientific, Waltham, MA, USA) and then submitted to library preparation utilizing the Nextera XT kit (Illumina, San Diego, CA, USA).

### Setting genome sequencing assembly

The whole genome sequences of *S. aureus* RMI-014804 used the MiSeq sequencing technology from Illumina with 2 × 150 bp to obtain its results. Poor and low-quality reads ends are filtered and trimmed using Trimmomatic and then assembled using SPAdes v3.5.0 [26]. To predict tRNA, rRNA, and protein-coding genes (CDS), ARAGORN v1.2.34 [27], RNAmmer v1.2 [28], and Prodigal v2.60 [29] were utilized, respectively. BLAST is then used for database searches and sequence comparisons [30].

### Genome annotation

The final draft genome sequence of *S. aureus* RMI-014804 was utilized for annotation using RAST (Rapid Annotation using Subsystem Technology) [31]. For the identification of genes involved in adhesion and biofilm formation, RAST platform and the VFDB (Virulence Factors of Pathogenic Bacteria) reference database were used [32]. For the annotation of antimicrobial resistance genes and toxin genes, ResFinder v4.1 and VirulenceFinder v2.0 tools from the Centre of Genomic Epidemiology, were used, respectively [33]. To standardize the analyses, all genomes were annotated with Prokka v1.14.6 [34]. Then these annotated genes were manually compared for genomic characteristics after being exported from the RAST server via an Excel table. The CGView server generated a graphical representation of the genomes' circular map [35].

### Phylogenetic analysis

The phylogenetic analysis of the *mecA* gene from different species of *S. aureus* that cause hospital infections was retrieved from the National Center for Biotechnology Information (NCBI) database. The phylogenetic tree was built using the neighbor-joining method as performed in MEGA X [36].

### Sequence data availability

The genome project has been filed publically with the NCBI under accession: SAMN19915631, BioProject: PRJNA741883, and BioSample: SAMN19915631 [37].

## Results

### Genome project history

The whole genome sequence of *S. aureus* RMI-014804 was deposited in the NCBI (http://www.ncbi.nlm.nih.gov) database. The result is given in Table 1.

### Specifications of the genome

The RMI-014804 strain's genome is 2,821,361 bp long, with an average G + C content of 28.76% and only has one main circular chromosome. The genome is expected to include 2,022 genes, including 1,962 protein-coding genes, 60 RNA genes, 1 CRISPR repeat, 15 rRNAs, 44 tRNAs, one tmRNA, and 0 pseudogenes. Table 2 [38-52] shows the classifications and general characteristics of *S. aureus* RMI-014804. The genome of the *S. aureus* strain RMI-014804 is described in Table 3.

### Genome annotation

**Fig. 1.**
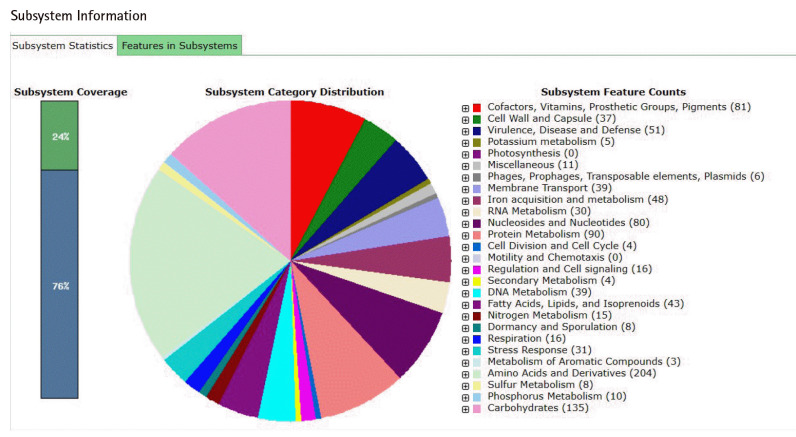
Summary of annotation of RMI-014804 strain using RAST substystem.

**Fig. 2.**
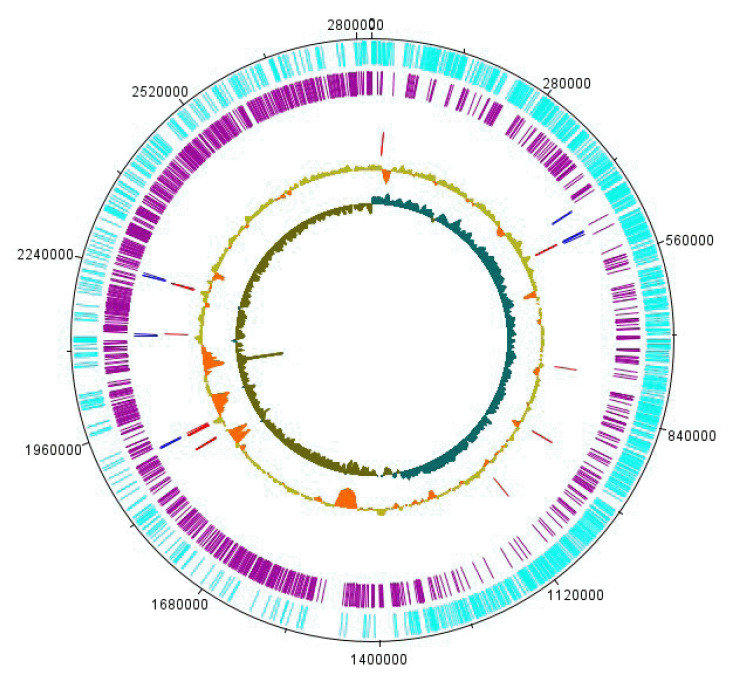
The genome is represented graphically as a circular map. From the outer to the inside. Forward strand genes (cyan), reverse strand genes (purple), RNA genes (tRNAs, blue; rRNA, red), GC concentration, GC skew.

The annotation of the whole genome of *S. aureus* strain RMI-014804 on the RAST server (Fig. 1) showed a total of 2,022 genes belonging to 254 subsystems including cofactors, vitamins, prosthetic groups, pigments, cell wall and capsule and virulence, disease and defense, only one main circular chromosome. The graphical circular maps of the RMI-014804 genome are shown in Fig. 2.

### Genes involved in virulence, disease, and defense

The results showed that 51 genes were responsible for virulence, disease, and defense, 21 genes for adhesion, four genes for bacteriocins, ribosomally synthesized antibacterial peptides, 17 genes for antibiotic resistances and toxic compounds, and nine genes for invasion and intracellular resistance. Some of the functional proteins encoded by these genes are clumping factors A and B, chaperone, fibronectin binding protein, collagen binding protein, two-component response regulator BceR and YvcP, acetyl-coenzyme A carboxyl transferase alpha and beta chain, mercuric reductase, thioredoxin reductase, fosfomycin and fluoroquinolones resistance, MerR family and multidrug resistance protein.

### Phages, prophages, transposable elements, and plasmids

The results showed that six genes encode for phages, prophages, transposable elements, and plasmids, with five of them responsible for phages, prophages, and pathogenicity islands and one for plasmid-related functions.

### Phylogenetic analysis of strain RMI-014804 using *mecA* gene

**Fig. 3.**
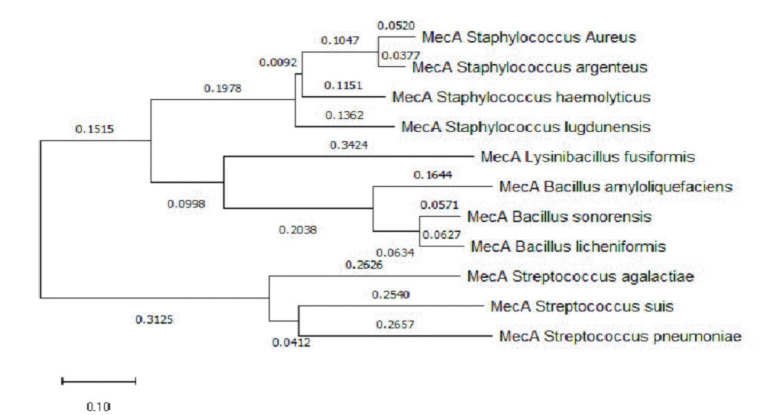
A phylogenetic study of the mecA gene from several organisms that cause hospital infections is shown in the figure. The neighbor-joining method was used to build the phylogenetic tree. The computations to calculate phylogenetic distances were performed using MEGA v9.

We chose to use the *mecA* gene for sequence similarity analysis instead of the 16s rRNA gene because the *mecA* gene is specific to staphylococci and is known to be a useful marker for detecting methicillin-resistant strains [53]. We wanted to focus on the genetic relatedness of methicillin-resistant staphylococci rather than the overall relatedness of bacterial species. The phylogenetic analysis of the *mecA* gene revealed that the RMI-014804 strain shares the most similarities with other *S. aureus* strains (Fig. 3).

### Analysis of the metabolic pathway

**Fig. 4.**
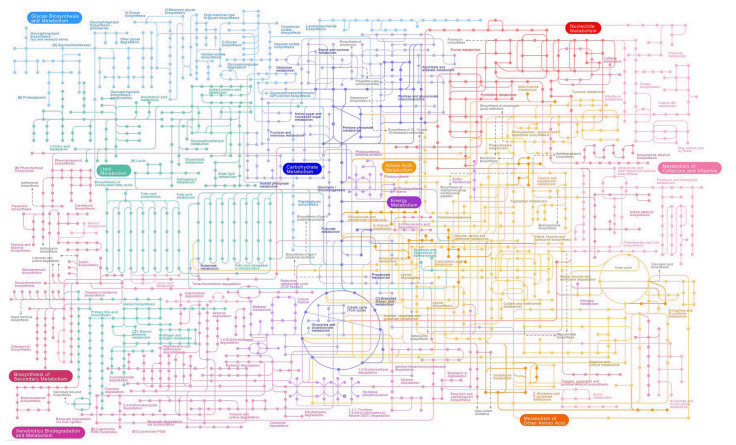
A diagram showing each metabolic pathway used by Staphylococcus aureus. Metabolites are represented by nodes, with forms reflecting the class of metabolites. Lines show reactions.

Based on annotated EC numbers and a custom enzyme name mapping file, Pathway Tools software version 26.0 was used computationally to generate the metabolic pathway/Genome Database (PGDB) as shown in Fig. 4 [54]. As with a Tier 3 BioCyc PGDB, the database has not been manually edited and may include mistakes (Table 4) [55].

## Discussion

The present research showed the genome sequence of *S. aureus* isolated from Peshawar, Pakistan to have phylogenetic allocation utilizing the *mecA* gene to indicate the bacteria's evolutionary relationships. The phylogenetic analysis of the entire *mecA* gene sequence of strain RMI-014804 revealed that the strain belongs to the genus *Staphylococcus*. The annotated complete genome sequence of the strain RMI-014804 was 2,821,361 bp long and contained 1,962 coding regions (CDS).

Whole genome sequencing data was also used to examine antibiotic resistance and pathogenicity mechanisms. The isolate's drug resistance could be caused by the bacteria's ability to accumulate multiple genes on resistance (R) plasmids coding for a single drug resistance within a single cell, or by increased expression of genes coding for multidrug efflux pumps, which extrude a wide range of drugs [56].

The present research showed that the strain was found to have a variety of resistance mechanism including the use of resistant genes TcaR, TcaA, TcaB, TetR, PBP2a, or secretion of enzymes (DNA gyrase subunit A, DNA gyrase subunit B, topoisomerase IV subunit A, topoisomerase IV subunit B, and beta-lactamase repressor) allowing it to use the efflux pump mechanism. The genome of RMI-014804 contains virulence genes (*scn*, *hlgA*, *hlgB*, *hlgC*, *splA*, and *splB*), enterotoxin genes (*sed*, *sei*, *sej*, tst, and *lukE*), and antibiotic resistance genes (*mecA*, *NorA*, *NorB*, *NorC*, MgrA, MepR tet(K), dfrG, and blaZ). We also revealed Fosfomycin resistance gene and chromosomal mutations in the ciprofloxacin resistance genes *gyrA* and *grlA* [57].

Six potential MarR family transcriptional regulators were also found in the RMI-014804 genome. These were identified as a highly conserved group of multiple antibiotic resistance regulators that respond to a broad range of drugs [58]. The presence of *mecA* gene, which is located in the staphylococcal cassette chromosome mec (SCCmec) element, encodes a penicillin-binding protein (PBP2a) with a lower affinity for b-lactams, is responsible for the methicillin-resistant *S. aureus* phenotype. Methicillin resistance-related proteins (*FemC*, *FemD*, *FmtA*, and *FmtB*) are also found in the isolated strain. Such a result should be taken into account while developing an effective therapy platform. The antibiotic-resistant genes of RMI-014804 showed that this strain is complicated and has a broad spectrum of cross-antibiotic resistance.

Whole genome sequencing may eventually displace a wide range of diagnostic and reference testing. Whole genome sequencing enables us to explore the epidemiology and genomic repertoire of *S. aureus* in clinical settings, which also gave proof of the organism's frequently underrated complexity. The current study provides information about an important antibiotic-resistant bacteria strain. The *S. aureus* RMI-014804 is resistant to tetracycline, fluoroquinolones, quinolones, acriflavin, penicillin, piperacillin, amoxicillin, ampicillin, and trimethoprim/ sulfamethoxazole. This result clearly suggests that other undiscovered determinants are directly or indirectly involved in the transcriptional regulation of *S. aureus*. The whole genome of the RMI-014804 strain can give insight into the genetic basis of virulence, antibiotic resistance, and phages of *S. aureus* and could lead to a better understanding of its pathogenesis and the development of new strategies to prevent the spread of staphylococcal infections.

## Figures and Tables

**Table 1.** Genome project information

Property	RMI-014804
Isolation	Sputum
Geographic location	Peshawar, KPK, Pakistan
Sample collection time	2,020
Sequence platform	Illumina MiSeq
Libraries used	2 × 150 bp
Assembly method	SPAdes v. 3.9.0
Date of release	2021-06-28
BioProject	PRJNA741883
BioSample	SAMN19915631
Locus tag	KUE46
Relevance	Medical

**Table 2.** *Staphylococcus aureus* strain RMI-014804 classification and general characteristics according to MIGS guidelines [38]

MIGS ID	Property	Term	Evidence code
	Classification	Domain *Bacteria*	TAS [39]
		Phylum *Firmicutes*	TAS [40]
		Class *Bacilli*	TAS [41]
		Order *Bacillales*	TAS [41]
		Family *Staphylococcaceae*	TAS [41]
		Genus *Staphylococcus*	TAS [41,42]
		Species *Staphylococcus aureus*	TAS [41]
		Strain RMI-014804	
	Gram strain	Positive	TAS [16,43,44]
	Cell shape	Rounded	TAS [44]
	Mortality	Non-motile	TAS [43,44]
	Sporulation	Non-sporulating	TAS [43]
	Temperature range	Mesophile	TAS [45]
	Optimum temperature	37°C	TAS [45]
	Salinity	Not reported	NAS
MIGS-22	Oxygen requirement	Aerobic and facultatively anaerobic	TAS [16,44]
	Carbon source	Glucose	TAS [46,47]
MIGS-6	Habitat	Host	TAS [42,48]
MIGS-15	Biotic relationship	Intracellular facultative pathogen	TAS [49,50]
MIGS-14	Pathogenicity	Human, poultry, cattle, birds, and goat/sheep	TAS [51,52]
	Isolation	Sputum	IDA
MIGS-4	Geographic location	Peshawar, KPK, Pakistan	IDA
MIGS-5	Sample collection time	2020	IDA
MIGS-4.1	Latitude	34.008	
MIGS-4.2	Longitude	71.57849	
MIGS-4.3	Depth	Not reported	
MIGS-4.4	Altitude	Not reported	

Evidence codes: IDA, Inferred from Direct Assay (first time in publication); TAS, Traceable Author Statement (i.e., a direct report exists in the literature); NAS, non-traceable author statement (i.e., not directly observed for the living, isolated sample, but based on a generally accepted property for the species, or anecdotal evidence). These evidence codes are from the Gene Ontology project [52]. If the evidence code is IDA, then the property was directly observed for a living isolate by one of the authors or an expert mentioned in the acknowledgments.

**Table 3.** Genome statistics of the newly identified strain

Attribute	Value	% of Total
Genome size	2,821,361	100
DNA coding region (bp)	1,521,337	53.29
DNA G+C content	406,116 + 405,281	28.76
Total genes	2,022	100
RNA genes	60	2.97
rRNA operons	15	0.74
Protein-coding genes	1,962	97.03
Pseudogenes	0	
CRISPR repeats	1	

**Table 4.** Metabolic network analysis

Attribute	Value
Total genes	2,022
Enzymes	687
Enzymatic reactions	1,265
Metabolic pathways	186
Transport reactions	76
Protein complex	66
Transporters	200
Compounds	944
